# Future Developments in Geographical Agent‐Based Models: Challenges and Opportunities

**DOI:** 10.1111/gean.12267

**Published:** 2020-12-04

**Authors:** Alison Heppenstall, Andrew Crooks, Nick Malleson, Ed Manley, Jiaqi Ge, Michael Batty

**Affiliations:** ^1^ School of Geography University of Leeds Leeds U.K.; ^2^ Alan Turing Institute The British Library London U.K.; ^3^ Department of Computational and Data Sciences and Department of Geography and Geoinformation Science George Mason University Fairfax VA USA; ^4^ Centre for Advanced Spatial Analysis (CASA) University College London London U.K.

## Abstract

Despite reaching a point of acceptance as a research tool across the geographical and social sciences, there remain significant methodological challenges for agent‐based models. These include recognizing and simulating emergent phenomena, agent representation, construction of behavioral rules, and calibration and validation. While advances in individual‐level data and computing power have opened up new research avenues, they have also brought with them a new set of challenges. This article reviews some of the challenges that the field has faced, the opportunities available to advance the state‐of‐the‐art, and the outlook for the field over the next decade. We argue that although agent‐based models continue to have enormous promise as a means of developing dynamic spatial simulations, the field needs to fully embrace the potential offered by approaches from machine learning to allow us to fully broaden and deepen our understanding of geographical systems.

## Introduction

Individual‐based methods, in particular agent‐based models, have seen a rapid uptake by researchers across the social and geographical sciences in the past 20 years (Macal 2016). Agent‐based models were first formally proposed in the early 1990s (e.g., Epstein and Axtell 1996) but their lineage goes back much further to the development of models of individual locational decision‐making in the 1950s and 1960s in the influential work of Hagerstrand (1953), Donnelly et al. (1964), and Schelling (1969) among others. They are now reaching a point of acceptance as a research tool across the geographical and social sciences, exploring such phenomena as epidemiology (Shook and Wang 2015), invasive species (Anderson and Dragićević 2020), settlement patterns (Bura et al. 1996), and segregation (Benenson and Hatna 2011) (see Polhill et al. 2019 for further discussion on the applications of ABMs and their use in policy).

However, there remain significant methodological challenges, for example, in: recognizing and simulating emergent phenomena; agent representation; construction of behavioral rules; calibration and validation (see Axelrod 2007; Torrens 2010; Filatova et al. 2013; Lee et al. 2015). Axelrod (2007) listed a number of challenges facing the modern agent‐based modeler which Crooks, Castle, and Batty (2008) extended. These challenges were presented at a time when agent‐based modeling was beginning to garner real excitement in the social sciences because of its potential to simulate individual behavior and actions. The increase in individual‐level data to enrich such models, advances in computing power, and improvements in agent‐based modeling software libraries all presented possible solutions to some of the original challenges put forward. However, while some major questions remain unresolved, advances in data and computing have brought with them a new set of challenges. This article will review some of the challenges that the field has faced, the opportunities available to advance the state‐of‐the‐art, and the outlook for the discipline over the next decade. To begin, we briefly summarize some of the recent important developments that have been driving the research in this area.

One of the major forces behind the increased interest in agent‐based modeling can be attributed to a notable shift in the information technologies and computing resources that are available. Within the spatial sciences this has resulted in a shift from models that simulate at the aggregate level to those that can capture local dynamics and interactions (Batty 2008). Much of the focus of research is now on the role of the individual and how decisions and behaviors play out over space and time. This change in thinking has been neatly articulated by O’Sullivan et al. (2012) and Batty (2013) who both emphasized the idea that social systems, and cities in particular, are driven by human decisions and the networks that exist between individuals. To be able to understand and explore spatial and social systems, an approach that places emphasis on the individual entities (e.g., humans, firms, or agencies) is clearly required. This is where agent‐based modeling comes in; its ready ability to represent heterogeneous decision‐makers and their interactions has made it a mainstream approach within the discipline of geography.

From the initial trickle of exploratory agent‐based models with an explicit spatial focus (e.g., Sanders et al. 1997), there has been a rapid increase in the number of applications that range from simulating pedestrians over seconds to the rise and fall of cities over centuries (e.g., Pumain 2012; Torrens 2017). With the growth of agent‐based modeling, a number of position pieces have appeared that have both critically appraised the achievements of the field, but more importantly, set out an agenda of challenges that need to be overcome if the field of agent‐based modeling is to become more mainstream. By and large, these challenges are methodological and include model parameterization, establishing robust behavioral rules, and their calibration and validation (see recent papers by Elsawah et al. 2020; Filatova et al. 2013; Manson et al. 2020; O’Sullivan et al. 2016; Polhill et al. 2019 which discuss these challenges in more detail).

As with many modeling techniques, the “realism” of an agent‐based model is directly correlated to the richness of the data that are input. A key change in the past decade is the appearance of so called “big data,” a term that is widely used to describe large, often rich and messy new forms of data, for example, those from mobile phone operations, credit card payments, or from social media contributions (see Kitchin 2013 for a discussion). These data have the potential to enrich our models with a more robust view/insight into the drivers and processes than previous modeling efforts have been able to achieve; for example, the ability to obtain near real‐time information about the underlying system (e.g., Crols and Malleson 2019), or to potentially to answer the “last mile” question (e.g., Wise et al. 2018). Although the tools to interrogate these data and extract real value and insights are still limited, it is an active area of research (e.g., see Singleton and Arribas‐Bel 2019). However, the opportunities that these data offer are matched by the number of challenges that accompany them. For example, how can we readily link data sets together (wrangling) or analyze these data to detect important drivers and behaviors that emerge over space and time?

The recent interest in machine learning (ML) approaches can potentially offer tools that are better able to interrogate these new data than existing methods; consequently there is an increase of interest in using them within agent‐based models (see Abdulkareem et al. 2019; Heppenstall and Crooks 2019; Runck et al. 2019). In the following sections, we elaborate further on both the challenges faced by the field and the potential opportunities that other research areas such as big data and ML, offer agent‐based modeling. We end the discussion by offering a perspective on how new opportunities can be exploited in the development of geographical agent‐based models (Section “The future outlook”).

## Challenges and opportunities for agent‐based modeling

Rather than rework the multitude of challenges which have been identified elsewhere (see Crooks, Castle, and Batty 2008; Manson et al. 2020), we examine specific challenges and highlight some of the existing opportunities that can be leveraged to address them. These challenges range from the representation of agents at different spatial and temporal scales (Section “Representativeness and scale”), to the incorporation of behavior into models (Section “Behavior”), to issues of validation (Section “Validation”) through to questions about model uncertainty (Section “Uncertainty, emulators and ensembles”) and real‐time modeling (Section “Real‐time agent‐based modeling”). Interlinked with these challenges, we present a number of opportunities. Arguably the most significant ones are emerging through increases in computational power and data availability. These, in turn, have led to a surge in interest in multivariate methods such as machine learning (ML) as a means to make sense of the large volumes of messy data that are characteristic of novel “big” data sources. However, despite the growth in ML within geographical analysis, applications of ML to agent‐based modeling specifically are still sparse (see Crooks et al. 2020).

### Representativeness and scale

One of the most characteristic features of agent‐based models are the agents themselves. Within these models, agents have the ability to represent different entities (e.g., individuals, households, etc.) and their dynamics over different temporal and spatial scales. There is often little discussion about how to aggregate rules and behaviors from the individual level to groups while preserving the most important dynamics and details. Extracting aggregate trends and behaviors from the complex interactions of many agents broadens the explicability of a model to those working outside of agent‐based modeling research.

As agent‐based models have evolved from early abstract models (e.g., Schelling 1969) to more empirical ones with realistic spatial representations (e.g., Benenson et al. 2002), the spatial representativeness of the model becomes an important issue; this is the so‐called *scale* issue. For example, Evans and Kelley (2004) explored the scale dependence of empirical agent‐based models in land‐use and land‐cover change (LUCC) applications. The authors varied the spatial resolution of the landscape in agent‐based models where agents make decisions, and where the input data (on land‐use change and topography) are used to calibrate the model. By doing so, Evans and Kelley (2004) found that the calibrated model parameters change as the spatial resolution of the model changes. The authors concluded that the choice of scale and spatial representativeness can affect the model outcomes and should be a particular concern in the design and application of agent‐based models.

An advantage of using an agent‐based modeling approach in a spatial setting is that it provides a flexible way of combining processes at multiple scales, from local to regional to national or even global (O'Sullivan 2008), potentially creating feedback across scales. For example, An, Grimm, and Turner (2005) developed an agent‐based model that integrates various processes, submodels and data at multiple scales, using a coarser resolution for submodels requiring extensive human demographic factors and a finer (90 m) resolution for submodels requiring finer spatial characteristics. The model also incorporated cross‐scale data from different sources, including remote sensing and satellite image data, high‐resolution biophysical data, elevation data from GPS, and sociodemographic data from census and surveys that cover a larger region. This multiscale approach to an agent‐based model allowed for a more flexible and heterogeneous spatial representativeness. That the agent‐based model can incorporate data at different scales is useful in the calibration and validation of the model, especially in pattern‐orientated modeling that matches empirical data with model outcomes across multiple scales (Grimm et al. 2005).

Related to the scale issue is the modeling of individual agents and groups. While agent‐based models can represent different entities (individuals and households) at the individual level, there is often little discussion on how to aggregate individual agent behavior into groups while preserving the most important dynamics and details. One potential approach is to define a typology for agents in the model. Valbuena, Verburg, and Bregt (2008) describe a bottom‐up, empirical approach that defines an agent typology for farmers in the Netherlands. They identified and parameterized five different agent types based on farmers’ views, farm characteristics, and land‐use decisions, and strategies. Having a manageable number of agent typologies broadens the model’s explicability to those working outside of agent‐based modeling research. It also creates a direct connection between the agent‐based model and behavioral and social theories based on different social groups. The main difference between an agent‐based model with an agent typology and an equation‐based model with heterogeneous representative agents is that the agent‐based model allows both interactions between and within agent types, with agents of the same type not acting as one.

So how can we exploit new opportunities to represent heterogeneous agents and their dynamics? ML can be used to inform agent representativeness by defining agent types from the bottom‐up using empirical data. Clustering methods can be used to identify agent groups or typologies from agent characteristics, behaviors, and spatial or environmental attributes. ML can be especially helpful when the data associated with the agents are plentiful and are sourced from multiple sources (as is the case with “big” data), but the theoretical underpinning of the agent typology is absent. One example is work by Valbuena, Verburg, and Bregt (2008), which used decision trees to classify different agent types in farmers in the Netherlands or the work of van der Zanden et al. (2016) who used clustering analysis to classify agricultural landscape typology in Europe. Van der Zanden et al. (2016) compared the bottom‐up, data‐driven typology using ML methods with the top‐down, expert‐based approach, and concluded that the expert‐based approach has the advantage of transparency and interpretability while the ML approach has the advantage of avoiding bias and discovering unknown patterns in the data. However, the authors point out that an issue with the ML approach to agent typology is that it is hard to transfer the typology to another data set to represent future conditions and regime shifts. The lack of interpretability and transferability of ML methods remains a challenge for its use in agent‐based modeling, as well as more generally.

In terms of integrating processes at different spatial scales with possible cross‐scale feedbacks, agent‐based models are highly flexible. They can use cross‐scale data for calibration and validation, which could be very useful given the increasing availability of non‐numeric data (e.g., images and videos) and individual‐level data. For example, spatial and temporal data received from remote sensors can be used to inform and improve the spatial representation of agent‐based models using deep learning methods (Oloo 2019). ML can also be used to calibrate individual agent behavior, and then, cross‐validate the model with aggregate data. For example, Zhang et al. (2016) adopted a data‐driven approach to agent‐based modeling and used Maximum Likelihood Estimation to calibrate individual decisions to adopt solar panels in San Diego, California. They then cross‐validated the model with aggregated adoption rates across geographic regions.

### Behavior

Representing and simulating a range of different behaviors is one of the most enticing aspects of agent‐based modeling. However, one of the key challenges within this area lies in identifying important behaviors at an adequate granularity to fully capture individual behavior and decisions over different spatiotemporal scales. Another challenge relates to the issue that many of the rule sets that are constructed and embedded within agent‐based models can only ever support relatively simplistic behavior (e.g., move to a nearest location where your preferences are met as in the Schelling (1969) model). For applications where the behavior is not well understood, there is a danger that the key drivers of the system under consideration are overlooked.

There are a wide variety of methods for embedding behavior, ranging from simple probabilistic rules to more advanced behavioral frameworks (such as BDI (Beliefs Desires Intentions architectures (Bratman, Israel, and Pollack 1988) and PECS (Physical conditions‐Emotional state‐Cognitive capabilities‐Social status) reference model (Urban 2000)). However, modelers rarely consider alternative behavioral frameworks or explain why one was chosen over another (see also An 2012; Filatova et al. 2013; Balke and Gilbert 2014; Groeneveld et al. 2017; Schlüter et al. 2017). Two important developments might foster a greater recognition of the importance of robust cognitive models for some applications. First, efforts are being made to develop cognitive frameworks within modeling packages, such as the BDI framework in MATSim (Horni, Nagel, and Axhausen 2016) or (Taillandier, Therond, and Gaudou 2012). Second, frameworks such as ODD + D (Müller et al. 2013) and Modeling Human Behavior (Schlüter et al. 2017) which attempt to provide a means for communicating and comparing different theories of individual human decision‐making are showing promise. The development of standard tools for coding human behavior into cognitive frameworks does not seem unreasonable and would improve the realism within agent‐based models (to the extent that these theories are correct).

This is also an area where new forms of data assisted by data science and machine learning can play an important role. There is an ever increasing number and diversity of new data sets that can give new and deeper insights into key behaviors over space and time. These include movement and point of interest databases such as SafeGraph (2020), mobility trajectories (e.g., Zheng et al. 2009) or other app derived mobility patterns (e.g., Cuebiq, Unacast), transport system use (e.g., travel cards), cell phone and social check‐in data of individual movement, and communication or online reviews such as Yelp and Trip Advisor. While such work has yielded insights into movement or what people value about places (e.g., Malleson et al. 2018; Yang et al. 2019; Yuan, Crooks, and Züfle 2020), there are issues with such data ranging from confidentiality, representativeness, and usage rights.

As the size of new data sets grow, machine learning (ML) is beginning to make a necessary appearance. ML offers the potential to improve the representativeness (and assumptions) of agents through both identification of behavior in data, the creation of more realistic rule sets, and also in extending agents’ adaptability through learning during simulations. Examples of ML being used include “filling in the gaps” in behavioral data sets (Abdulkareem et al. 2019), to create a representation of an agent’s mental models using natural language processing and Bayesian learning (Runck et al. 2019), and to optimize behavioral strategies using reinforcement learning (Bone, Dragicevic, and White 2011).

There are a number of data science approaches that are being used to inform behavioral rules within agent‐based models. However, many of these approaches are not new although “early” examples are quite sparse: for example, Heppenstall, Evans, and Birkin (2007) used a genetic algorithm (GA) to search for optimal parameters for behavior rules within an agent‐based model, while Soman et al. (2008) combined a GA with an agent‐based model to simulate landowner behaviors. It is only within the past few years, with increases in data and algorithms that ML approaches are beginning to be discussed within the literature. The majority of the contributions within this area are constrained to theoretical or conceptual contributions that discuss the potential of ML, with few empirical examples available.

Recurrent neural networks (RNNs) have been a popular option for time series forecasting, with many applications ranging from modeling COVID‐19 transmission (Chimmula and Zhang 2020) to upcoming earthquake prediction (Kaggle 2020). However, there are some examples involving individual behavior; Jäger (2019) used a neural network to simulate the decision‐making process within an agent‐based model while Deng and Chen (2019) used the same approach to simulate the behavior of office workers. Another area that is beginning to show promise for simulating behavior is Bayesian modeling (Congdon 2007). For example, Abdulkareem et al. (2019) used Bayesian networks and survey data to explore the spread of cholera in Kumasi, Ghana. Specifically, they used Bayesian networks with respect to improving risk perception and decision‐making about where to get water during a cholera outbreak. In some cases the behavior of the agents can also be derived from data. For example, Zhang et al. (2016) demonstrated the use of machine learning techniques (regression in this case) to represent the behavior of the agents in their model of solar panel adoption. Although such an approach assumes that the behaviors are simple enough to be approximated by a regression model, it offers an exciting opportunity for defining behavior in the absence of a well validated rule‐based behavioral model.

One area within ML that is gaining traction is that of reinforcement learning which can be considered as goal‐orientated learning through iterative interactions with the environment (Dhalke et al. 2020; Wooldridge 2020). There are few examples within the spatial context (notably Sert et al’s (2020) RL application to segregation dynamics), but a promising route forward could be allowing the agents to learn their 3D environment for themselves. Through methods such as deep reinforcement learning, where positive behaviors are learned through a repeated exposure to an environment, or deep neural networks, which capture uncertainty and incomplete knowledge representations along with large‐scale individual tracking data, it may be possible to teach agents how to navigate spaces as if they were human. These “spatial learning” agents may both better reflect the actual behaviors of humans and model their behavior under changing conditions. Only a handful of simulation models have sought to integrate aspects of spatial cognition and bounded learning (Manley, Orr, and Cheng 2015; Manley and Cheng 2018). However, progress is rapidly being made that replicates cognitive processes of agent behavior (e.g., Banino et al. 2018) social interactions (e.g., Leibo et al. 2017), and autonomous vehicle interaction with 3D spaces (e.g., Shah et al. 2018), but integration into geographical modeling remains an opportunity.

### Validation

The growth in available data, particularly high‐resolution temporal data, coupled with increasing computing power, is fostering the development of innovative methods to tackle one of the most difficult and controversial aspects of agent‐based modeling: namely validation. Validation is the process of estimating how well a model is able to reproduce the behavior of the target system. In some cases, particularly when hypothetical scenarios are under examination, strict validation with external data may not be necessary. Indeed it has been argued (Oreskes, Shrader‐Frechette, and Belitz 1994) that it is *impossible* to validate numerical models of open systems in the first place. External, unknown parameters and hidden variables that cannot be accounted for by a model will usually influence the behavior of the system under study, so it would be somewhat “suspicious” (Polhill and Salt 2017) if an agent‐based model were able to match observed data perfectly. Nevertheless, some form of validation is often essential and has been a major challenge for agent‐based modelers for years (e.g., Crooks, Castle, and Batty 2008). As Heppenstall and Malleson (2020) comment: *“the validation of agent‐based models remains a dark art at worst and haphazard at best.”*

The challenge that sets agent‐based modeling aside from other forms of modeling stems from the fact that agent‐based models simulate heterogeneous individuals that evolve over space and time. Thus, interdependencies between variables, feedback, and new information via emergence are difficult, if not impossible, to observe in reality (Batty and Torrens 2005). Fortunately, some “big” data sources offer the potential to validate behaviors directly, or at least over very fine spatial/temporal scales, but finding appropriately rich and detailed data to validate such systems is still difficult and we cannot simply assume that as individual‐level data become richer in detail this issue will be easily resolved. There are in fact in agent‐based models many decision processes that define the way individuals behave that cannot be easily observed and are often assumed, thus, making it impossible for any comprehensive kind of validation to take place.

However, assuming appropriate data are available, good progress has been made toward providing tools and knowledge that can assist agent‐based modelers with the statistical and technical aspects of validation. Related to validation, Thiele, Kurth, and Grimm (2014) have published a comprehensive “cookbook” that demonstrates a range of advanced statistical methods for parameter estimation and sensitivity analysis of agent‐based models. Furthermore, the use of relatively advanced methods such as Approximate Bayesian Computation (van der Vaart et al. 2015) allow prior information to be included in the model estimation process which provides a more nuanced treatment of the uncertainty of the model and its parameters as well as potentially greater efficiency than typical simulated minimum distance techniques (Grazzini, Richiardi, and Tsionas 2017).

The difficulties associated with validating agent‐based models can be summarized as:

(i) The nature of the underlying system—agent‐based models are typically built to simulate complex, emergent, and open systems that will inevitably include unknown parameters;
(ii) The nature of an agent‐based model—the interdependencies between agents and their environments and the emergence of phenomena at different spatiotemporal scales mean that many aspects of the model are impossible to observe in reality;
(iii) Data availability—even if it were possible to observe a particular aspect of the system, it is unlikely that fine‐grained spatiotemporal data will be available to validate the behaviors of the agents directly.

The implications of (i) and (ii) above mean that validation by fitting to data may not by itself be a helpful measure of the “success” or “usefulness” of a model. An alternative to validation by fitting to data could be to examine the *ontological structure* of the model (Polhill and Salt 2017). Agent‐based models are somewhat unique, at least when compared to typical machine learning methods, in that the structure may give insight into the underlying system. This is not dissimilar to the idea of *face validation*, whereby modelers and others examine the model, its behavior and its outcomes to determine whether it *looks correct*, a principle advocated for simulation many years ago by Mandelbrot (1983). Domain experts can also be engaged to provide “expert involvement”—Hassan et al. (2013) present detailed recommendations for effectively using expert advice. In effect, the nature of agent‐based modeling makes it amenable to a variety of different “validation” methods.

Although it can be difficult, validation by fit‐to‐data can be nevertheless be a useful benchmarking process to estimate the “empirical adequacy” of a model (Oreskes 1994). For others, validation is seen as essential as a means of establishing the reliability of agent‐based modeling, without which its usefulness is limited (An et al. 2020). Pattern‐oriented modeling (Grimm et al. 2005) presents a framework for comparing agent‐based models to data at different spatiotemporal scales, but traditionally data at fine spatiotemporal scales have been hard to come by. Fortunately, the emergence of “big” data sources offers new opportunities for validation across various spatiotemporal scales and at levels of resolution that were previously not possible. Of course there are numerous issues with “big” data sources, particularly issues of bias and, privacy, but if a useful and reliable signal can be uncovered, then, there are potentially useful insights that can be gleaned. Here, machine learning will undoubtedly play a useful role by helping to simplify data through classification or by infilling missing data to attempt to resolve biases. As an example, Kavak et al. (2018) used Twitter data to estimate mobility behavior, creating agents and their behaviors directly from the tweets themselves, where the ultimate aim was to develop machine learning algorithms to drive the behavior of the agents directly. Alternatively, the agent‐based model itself can be used as a means of adding further richness to data that are spatiotemporally accurate but lack this kind of variety. For example, near real‐time sensors that measure population flow rates have very limited information about the individuals themselves (which is essential to maintain privacy), but these data can be used to evaluate potential agent‐based models of routine urban activity, and thus, shed light on the potential sociodemographics and activities of visitors to town centers (Crols and Malleson 2019).

### Uncertainty, emulators, and ensembles

Although reliable validation helps to ensure that models are robustly simulating the target system, to achieve a level of credibility within policy arenas we need to be able to quantify the (un)certainty in the predictions made. One of the major difficulties in developing agent‐based models of real‐world systems relates to the use of data to reduce uncertainty. As with all models, uncertainty arises through measurement noise, the choice of parameter values, and the model structure (Ghahramani 2015). With agent‐based models, however, these problems are compounded because: (i) the agent’s behavior is often determined by parameter values that are unique to each agent, hence, the number of possible parameters to calibrate can be extremely large; (ii) data are not usually available at all spatiotemporal scales (e.g., for individual agent actions as well as the aggregate evolution of the system); and (iii) the underlying systems are often complex so that even a perfectly calibrated model will inevitably diverge from the underlying system (Heesterbeek et al. 2015) over relatively short time scales. Although great strides have been made in the development of efficient static calibration methods for agent‐based models (e.g., Thiele, Kurth, and Grimm 2014; van der Vaart et al. 2015) much less work considers the problem of model divergence (i.e., parameter recalibration and state estimation during model runtime). This makes it impossible to predict individual movements and consequence of decisions in *real‐time* using agent‐based models. Although there have been preliminary attempts at developing real‐time agent‐based models, the need for more robust methods is great and there are many challenges that need to be overcome first (Tao and Qi 2019; Heppenstall and Malleson 2020; Swarup and Mortveit 2020). However, this is an area where there have been recent advances drawing on work from other disciplines.

Correctly quantifying the uncertainty associated with agent‐based models is essential if they are to become more mainstream and useful in policy. It is important to understand how uncertainty propagates through an agent‐based model so that we are able to (i) properly quantify the error associated with model outputs and; (ii) identify the most important sources of uncertainty so that they might be reduced. Here, the use of “ensemble modeling” could be advantageous, in which a number of instances of the same (probabilistic and agent‐based) model are executed simultaneously. As such models are defined to be probabilistic in terms of their predictive outcomes; they naturally diverge during the course of a simulation. By analyzing the range of model results across an ensemble of models, it is possible to begin to better understand how uncertain the outputs are. For example, if most models are broadly in agreement with respect to a particular result, then, it is possible to be reasonably certain that that result is a likely outcome. In the cases where the models in the ensemble are in disagreement about a result, then, there is more uncertainty. By presenting results from models alongside their uncertainty, modelers have an opportunity to more rigorously represent the reliability of their predictions.

In addition to pure ensemble methods, techniques developed in the field of Uncertainty Quantification (UQ) can help to quantify the uncertainty associated with complex numerical models. There are therefore considerable opportunities to adapt methods from UQ to better understand the propagation of uncertainty in agent‐based modeling. However, UQ are typically ensemble methods so, as discussed above, agent‐based modelers will need to run large numbers of models. This might be impossible for computationally expensive agent‐based models. Therefore, another opportunity for agent‐based models is to work on the development of efficient “*surrogates*” or “*emulators*” of the otherwise expensive models. One potential emulator is the Gaussian Process Emulator (see Bastos and O’Hagan 2009) because the emulator does not force any requirements on the structure of the underlying model and it also quantifies the uncertainty associated with their estimates. Other machine learning techniques for emulators, such as Neural and Bayesian Networks (Farah et al. 2014; Shrestha, Kayastha, and Solomatine 2009) have also shown promise. Most emulators of agent‐based models that have been developed to date use Gaussian Processes and they are typically developed to support calibration and sensitivity analysis (e.g., Dosi et al. 2018). If a robust emulator can be created for agent‐based models, then, a wealth of valuable uncertainty quantification techniques become available, not to mention the opportunities for more efficient sensitivity analysis and calibration.

### Real‐time agent‐based modeling

The emergence of new, “big” data sources has not only allowed agent‐based modelers to consider new approaches to the specification, validation, and calibration of models, but has also fostered approaches that are more strongly directed by the underlying observational data and can potentially be leveraged to reduce uncertainty in *real time*. The concept of Data‐Driven Agent‐Based Modeling (DDABM) refers to a suite of methods that are able to enhance or refine a model, making strong use of available observational data. This is particularly important for modeling systems where the underlying behavioral models are relatively well understood, but data are required to adequately represent all other aspects in the model. For example, Venkatramanan et al. (2018) present a data‐driven approach to build a model of infectious disease spread. The authors use a relatively simple behavioral model because the mechanisms of disease are well understood, but use empirical data to synthesize the population, initialize the social contact network, and calibrate the model parameters. For a comprehensive recent review of further data‐driven approaches see Kavak et al. (2018).

A related issue that even data‐driven models must face, and results as a natural consequence of the uncertainty, is that of model divergence (i.e., the model output diverging from the real data). Although this problem is less acute for models that explore phenomena over longer periods of time where some error will be averaged out, such uncertainty can be catastrophic for the analysis of scenarios that develop rapidly and depend heavily on the current state of the real system. Fortunately, methods exist in other fields that allow new data to be incorporated into models in real time (i.e., while the model is running) such as *data assimilation* (Lewis, Lakshmivarahan, and Dhall 2006). These offer considerable opportunities for agent‐based modeling.

Data assimilation refers to a suite of techniques that allow new observations from the real world to be incorporated into models (Lewis, Lakshmivarahan, and Dhall 2006). The techniques have largely evolved from fields such as meteorology and applications to agent‐based modeling are relatively rare, but some have begun to explore this area (e.g., Wang and Hu 2015; Ward, Evans, and Malleson 2016; Long and Hu 2017; Clay et al. 2020; Malleson et al. 2020). Although there are similarities, data assimilation is quite different from typical agent‐based parameter estimation. Parameter optimization does not consider the state of the model during runtime but rather searches for optimal parameter values once a simulation has finished. Rather than finding optimal parameter values (although they can do this as well), data assimilation techniques adapt the state of the model itself to try to bring it toward observational data.

The marriage of data assimilation methods and agent‐based models could be transformative for the ways that some systems are modeled. For example, a real‐time agent‐based model of disease spread could assimilate new data that are available in near real time and use them to reduce the uncertainty in a global model of disease transmission that runs continuously, making predictions about future disease clusters or enacting potential policy scenarios (Swarup and Mortveit 2020).

## The future outlook

Agent‐based modeling is a highly active and diverse field, enjoying wide success in a variety of contexts. The increasing availability of data, machine learning (ML) approaches, growing computational resources, deeper multidisciplinary thinking, and the simple intuition of the approach all suggest that agent‐based modeling as an approach has many promising future applications (see Waldrop 2018). Nevertheless, to achieve full maturity and credibility, agent‐based modeling for geographical applications requires focus in a number of areas. Perhaps the most pressing need is for a deeper focus on scientific method—specifically, replication, validation, and progression. It is in areas such as these, highlighted within our previous discussion, that there are clear opportunities for ML to move the discipline forward.

While ML is beginning to have an impact within agent‐based modeling, this is largely limited to discussion around its potential contribution using conceptual or theoretical rather than empirical examples. Research featuring ML tends to focus on the possibilities that ML offer, with little or no consideration about whether these approaches will contribute any new insights, or what the drawbacks of these approaches are. While ML potentially offers a set of new and exciting tools that *could* meet the methodological challenges of agent‐based modeling (e.g., calibration, validation, and realistic behavioral representation), there is a danger that ML, akin to “Big Data,” is simply a distraction from work on these issues. Moreover, ML is, to a large extent, simply an extension of multivariate methods to deal with big data that have their origins in basic statistical analysis, and which are still plagued by the limits to induction that all such analysis is subject to.

But what about the future? With increases in the volume and type of individual‐level data, there will be undoubtedly more research into ML approaches for identifying and classifying behavior. However, even with efforts through approaches such as microsimulation to create synthetic versions of individual‐level data sets, we will never possess the levels of data that we need to create fully robust agent‐based models. This is an area where ML methods, in particular the many varieties of neural networks, may fill in the gaps within data and discover new patterns of behavior over different spatial and temporal scales. These will in turn, improve the rigor and robustness of the validation that we can achieve. However, akin to agent‐based modeling, many ML methods are reliant on large amounts of detailed data; a more fruitful path may lie in facilitating ways for agents to learn and make decisions for themselves.

Probabilistic programing (Ghahramani 2015; Grazzini, Richiardi, and Tsionas 2017) and reinforcement learning (Dhalke et al. 2020; Wooldridge 2020) both offer mechanisms for improving behavioral responses, enabling agents to be more proactive than reactive. However, these approaches are largely untested, at least on real systems, and may not be appropriate for use with agent‐based models or with systems built from discrete, decision‐making entities in the first place. Hence, simpler systems are needed to develop and test these new methods, but these systems must exhibit some complex behavior (e.g., emergence, feedback loops, nonlinear behavior, etc.) and there must be sufficient data about them to allow their “true” state to be observed. To this end, perhaps biological systems such as *Physarum polycephalum*—“slime mould”—provide the ideal test bed. The rules that underpin their behavior are reasonably well understood, they exhibit complex outcomes, they can (and have been) observed in great detail, and they can be modeled reliably using agent‐based modeling (Jones 2015).

Finally, with the current appetite for creating “digital twins” of cities, we anticipate a rise in the adoption of Gaming engines, such as the Unity platform. Research in this area could bridge the gap from abstract representations of how individuals move and behavior around cities to real city simulations. But as the field is becoming more eclectic and as the quest for the integration of different model types continues, agent‐based models will increasingly become a feature of geographical models in general as they begin to influence the many dimensions as to how we articulate our systems of interest.
